# Distribution and Determinants of Non Communicable Diseases among Elderly Uyghur Ethnic Group in Xinjiang, China

**DOI:** 10.1371/journal.pone.0105536

**Published:** 2014-08-20

**Authors:** Lei Feng, Ping Li, Xihua Wang, Zhi Hu, Ying Ma, Weiming Tang, Yanli Ben, Tanmay Mahapatra, Xiaolin Cao, Sanchita Mahapatra, Min Ling, Anshuan Gou, Yanmei Wang, Jiangqin Xiao, Ming Hou, Xiuli Wang, Bo Lin, Faxing Wang

**Affiliations:** 1 Department of health service management, School of Health Service Administration, Anhui medical university, Hefei, Anhui, China; 2 Medical Department, The people’s hospital of Xinjiang Uighur autonomous region, Urumqi, Xinjiang, China; 3 Project-China, University of North Carolina, Guangzhou, China; 4 Department of STI Control, Guangdong Center for Skin Diseases and STI Control, Guangzhou, China; 5 Department of Epidemiology, Fielding School of Public Health, University of California Los Angeles, Los Angeles, California, United States of America; University Heart Center, Germany

## Abstract

**Background:**

Non-communicable diseases (NCDs) are showing an increasing trend globally as well as in China. Elderly population are more prone to these NCDs. Situation in China is worse owing to the higher proportion of geriatric population. Burden of NCDs and the role of their socio-demographic and behavioral predictors among these elderly and more so among the ethnic minority groups among them, need to be investigated specifically, owing to their distinct genetic background, lifestyles and behavior.

**Methods:**

A cross-sectional study was conducted among 1329 randomly selected persons of Uyghur ethnicity, aged 60 years or more in Xinjiang, the largest administrative division in China to measure the burden of NCDs, understand the distribution of socio-demographic, behavioral and life event-related potential correlates of them and to estimate the association of the NCDs with these correlates.

**Results:**

Among these participants 54.2% were female, 86.8% were married and more than half had only attended elementary school or less. 41.46% was suffering from at least one NCD. 20.22% had one NCD, 12.11% had two and 8.58% had three or more. 27.3% had hypertension, 4.06% had diabetes, 6.02% had hyperlipidemia, 7.37% had angina, 14.52% had cardiovascular diseases, 11.59% had any kind of cancers and 9.78% had chronic obstructive pulmonary diseases. Rural residents (OR = 1.45, 95% CI: 1.17–1.80, AOR = 2.00, 95% CI: 1.53–2.61) and current smokers had higher odds of having more NCDs (AOR = 1.53, 95% CI: 1.00–2.34). Additionally not being satisfied with current life, not being able to take care of self in daily life, currently not being involved in farm work, less intake of fresh vegetables, fruits and garlic, too less or too much salt intake, not having hobbies were found to be positively associated with having more NCDs.

**Conclusion:**

Implementation of effective intervention strategies to promote healthy life styles among the Uyghur elderly population of China seems urgent.

## Introduction

Non-communicable diseases (NCDs) have culminated into a major public health challenge worldwide, killing more than 35 million people each year and accounting for two-thirds of the cumulative global deaths [1]. Rapid urbanization, environmental pollution, economic transition and higher exposure to different behavioral risk factors like unhealthy diet, physical inactivity, addiction to tobacco and alcohol abuse have resulted into a progressive worldwide rise in the burden of NCDs and China, world’s most populous country, is no exception [2]–[4].

Over the past few decades, NCDs have emerged at a much faster rate in China than in western countries [2], [4]. As per the estimates provided by the Centers for Disease Control and Prevention (CDCs), about 82% of total disease burden was attributable to NCDs in China [5]. Compared to other leading G-20 countries, mortality rates due to NCDs like stroke, cancers, chronic obstructive pulmonary diseases (COPD) in this country were also quite high [6]. Based on the current trend, it is predicted that over next two decades, number of NCDs among people over 40 will become double or even triple in this country [6]. Evidences from recent surveys strongly suggested that the estimated prevalence of hypertension, high blood glucose, overweight/obesity and high blood cholesterol have increased alarmingly among Chinese adults [4], [6]–[11]. In a nationwide population based survey among residents aged 50 years or more, during 2010, self-reported prevalence of hypertension, diabetes, angina and stroke were estimated to be 27%, 7%, 8% and 3%, respectively [9].

It was estimated that during 2010, about 580 million citizens of China were exposed to at least one of the modifiable behavioral and nutritional risk factors known to be associated with NCDs [6]. There are current evidences in favor of a notable shift in the burden of disease from infectious to NCDs and gradually progressive aging of the population is probably worsening the scenario in this country [6], [9]. According to World Bank projections, in China there will be at least 40% increase in the burden of NCDs owing to this population aging by 2030 [6]. However, the NCDs services in the local area of China were very limited (community based clinics only could offer routinely hypertension test), particular among minority people like Uygur population. Hence a comprehensive understanding of the distribution and determinants of NCDs among elderly seems to be the need of the hour for designing effective public health interventions and prioritization of public health policies targeting those determinants so that the preventable burden of morbidity and mortality due to NCDs can be reduced substantially.

Although available evidences clearly suggested that burden of NCDs among elderly is growing rapidly in different regions of China resulting in serious social and economic consequences and the correlates of NCDs in this population may well be different than other age groups, efforts to identify definite action points for controlling the modifiable risk factors in this population have been slow and inadequate [3].

Xinjiang Autonomous Region is the largest administrative division of China and mainly inhabited by the Uyghur, one of the ethnic minority groups in this country [12]. Because of their different genetic backgrounds, customs, culture and food consumption, predictors of NCDs among elderly Uyghur might be quite distinct and evidences in this regard were limited [13].

Thus a detailed evaluation was called for to identify the distribution and determinants of NCDs among Uyghur elderly in Xinjiang, where alike other parts of the country, prevalence of NCDs also demonstrated a persistent upward trend during 1998 to 2008 and aging of population was identified as the main contributor [11], [14].

The objective of the current study was thus to measure the burden of NCDs, understand the distribution of their socio-demographic, behavioral and life event-related potential correlates and to estimate the association of the NCDs with these correlates among elderly population in Xinjiang Uyghur Autonomous Region of China.

## Methods

### Recruitment

This cross-sectional study was conducted, between the months of March and December, 2011. Stratified random sampling was used to select a diverse and representative sample of Uygur people in Xinjiang. Based on the ethnic, demographic, economic and cultural factors, Xinjiang was divided into three regions (southern, eastern and northern). In each of these regions, a specific unique identification number (UID) was assigned to every administrative unit. Next using a computer based random selection procedure, two UIDs were randomly chosen from the list of UIDs in each region. Thus, two units per region were selected randomly making a total of six overall sampling areas (Hotan, Lop, Hami, Huicheng, Urumqi County and Tianshan District). In each sampling area, the aforementioned random selection method was repeated, and one community/village was next selected randomly to have six randomly selected study sites altogether.

The inclusion criteria included 1) Age 60 years or more; 2) Uyghur ethnicity and 3) Agreeing to participate by providing written informed consent. From the population database (Chinese population registration system, which is maintained and held by the police department and can be assessed by submitting application form to the local/state government) of the residents of the selected communities, 2033 persons with Uyghur ethnicity who were aged 50 years or more were identified. Among them, 1766 had their residential address and other contact information correctly recorded in the population database. After contacting them, to minimize non-participation, all these 1766 seniors were screened by visiting them at their residence and 1455 of them met the inclusion criteria and 1329 agreed to participate and completed the survey.

### Structured Interview

A face-to-face interview using an interviewer-administered, structured questionnaire was conducted for each participant to collect information on demographics, behaviors, medical history and important events of life. Several PhD students who were trained for conducting the interviews using the same training modules and protocol conducted the interviews. In order to reduce the bias due to non-response, all the interviews were conducted at the residence of the participants.

The demographic information included age (continuous, and further categorized into 60–63/64–67/68–71/71–75/>75), gender (male/female), education level (elementary school or less/junior or senior high school/college and higher), marital status (never married/married/divorced or widowed), residency (urban/rural), occupation before retirement (officer/worker/farmer/others) and average annual income per person in the family [less than 2000 Yuan/2001–8000 Yuan/more than 8000 Yuan (while 1 US dollar = 6.05 Yuan)]. Ethnicity of each participant was determined based on the recorded ethnicity in the population registration system of China.

Behaviors were assessed by collecting information (yes/no) on smoking, alcohol drinking, religious belief, farm work and pesticide use. The participants were also asked whether they were satisfied with their current life (Very satisfied/satisfied/dis-satisfied/very much dis-satisfied), whether they had the ability to take care of themselves in daily life, what were their hobbies (watching TV, listening to radios, watching opera or films, reading books or papers, calligraphy, planting flowers, having pets, fishing, playing cards, playing Mahjong, playing chess, doing exercise, walking and travelling in a group). Negative events in the past two years were also enquired (significant deterioration in health status, serious economic difficulties, death of someone with intimate relation and important thing being lost or stolen). Besides these, we also asked the frequency of vegetables, fruits and garlic consumptions (two times or more per day/one time per day/less than one time per day but more than two times per week/one time per week or less). Average salt intake per day was estimated for each participant (3 grams or less per day/4–6 grams per day/7 grams or more per day). Chili consumption was enquired and categorized as often/sometimes/never.

### Disease assessment

Blood pressure of each of the participants was checked before and after the interview. In our study, hypertensives were defined as those who had prior diagnosis of hypertension or met any of following criteria 1) systolic blood pressures (SBP) in both readings being ≥140 mm of Hg; 2) the diastolic blood pressure (DBP) in both readings being ≥90 mmg.

Prior diagnosis of diabetes, hyperlipidemia, coronary heart disease/valvular heart disease (CHD), stroke, angina, cancers, and chronic obstructive pulmonary disease (COPD) were also enquired and recorded (yes/no). If the participant had any of the above mentioned NCDs, she/he was categorized as a subject having NCD. The total number of NCDs for each participant were calculated and categorized into four groups (0/1/2/≥3). Data on geriatric depression was also measured but findings were reported elsewhere (PONE-D-14-17995, Weiming Tang, “Burden and correlates of geriatric depression among Uygur elderly, observation from Xinjiang, China”).

### Data Analysis

Data was double-entered using the software EpiData 3.0 [15] and multiple logic checks were used to ensure the data quality. SAS version 9.1 [16] was used for all statistical analyses. Descriptive analyses were conducted to determine the distribution of the demographic factors, behaviors, life events and to calculate the prevalence proportions of different kind of NCDs. In addition, to assess the strength and direction of the association between NCDs and their potential correlates, ordinal logistic regressions were performed for univariate analysis [Odds ratio (OR) and 95% CI]. In the ordinal logistic regression, we categorized the participants into four groups based on the numbers of NCDs they had: having no NCDs/having one NCD/having two NCDs/having three or more NCDs. Since the principal dependent variable (number of NCDs) had 4 clearly defined categories (suffering from no NCD/1 NCD/2 NCDs/≥3 NCDs) with a typical order (0<1<2<3 or more) hence to have increased efficiency of analyses, to determine the association of the independent variables with the odds of having higher number of NCDs, we used ordinal logistic regression [17]. We further performed multivariate ordinal logistic regression adjusting for gender, education and occupation in model 1, and gender, education, occupation, age (continuous) and marital status in model 2. For each of the regression analyses we used appropriate model diagnostics and the models that we used did fit well (P<0.001) while the proportional odds assumptions for ordinal logistic regressions were also met in each of them.

### Ethics statement

The study process and content were approved by the Ethics Committee of Xinjiang Autonomous Region. Signed written informed consent was obtained from each participant prior to the interviews. Each of the participants had the discretion to freely decline or withdraw from this survey at any point of time. The filled-in questionnaires, written consent documents and computerized data were properly secured.

## Results

### Demographics and behaviors

**Figure 1:**
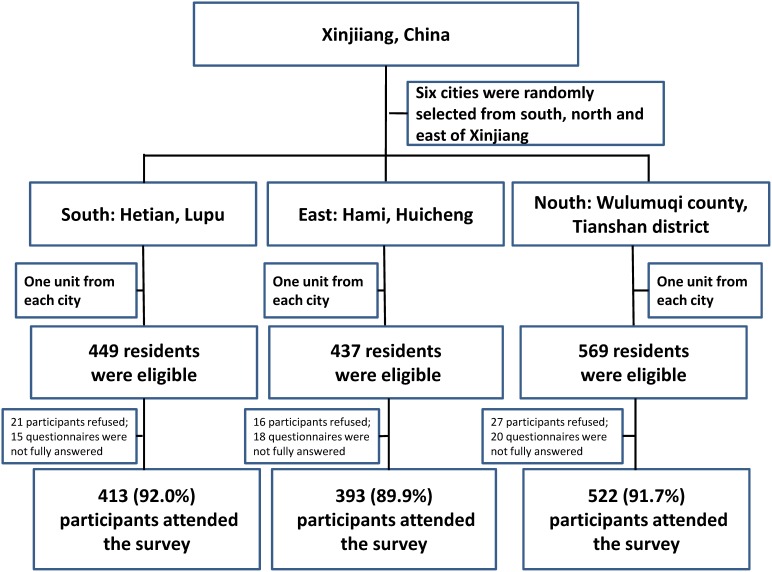
Flow Chart of the recruitment of the elderly Uyghur in Xinjiang Uyghur Autonomous Religion, China (N = 1329), 2011.

In this comprehensive cross-sectional study, overall 1329 elderly persons of Uyghur ethnicity were recruited in Xinjiang, China. Figure 1 presented the response rate in each selected region of Xinjiang. Among these participants, 720 were female (about 54.2%), 41% (42.6% among female and 37.2% among male) were aged between 60 and 63 years old, majority (86.8%) were married (82.5% and 91.8% among female and male respectively) and more than half had only attended elementary school or less (58.8% for female and 49.4% for male) (Table 1).

**Table 1:** Sociodemographic characteristics and prevalence of diseases among Uyghur elderly (60 or older) in Xinjiang Uyghur Autonomous Region, China (N = 1329), 2011.

Variables		Female (n = 720)			Male (n = 609)			Total (N = 1329)	
		Frequency	Percent	95% CI	Frequency	Percent	95% CI	Frequency	Percent
Age	*60–63*	307	42.64	39.02,46.26	226	37.23	33.38,41.09	533	40.17
	*64–67*	157	21.81	18.78,24.83	134	22.08	18.77,25.38	291	21.93
	*68–71*	103	14.31	11.74,16.87	94	15.49	12.60,18.37	197	14.85
	*72–75*	65	9.03	6.93,11.13	78	12.85	10.18,15.52	143	10.78
	*More than 75*	88	12.22	9.82,14.62	75	12.36	9.73,14.98	163	12.28
Residence	*Urban*	471	65.42	61.93,68.90	358	58.78	54.86,62.70	829	62.38
	*Rural*	249	34.58	31.10,38.06	251	41.22	37.29,45.14	500	37.62
Marital Status	*Married*	594	82.50	79.72,85.28	559	91.79	89.60,93.98	1153	86.76
	*Never married*	22	3.06	1.80,4.32	21	3.45	2.00,4.90	43	3.24
	*Divorced or widowed*	104	14.44	11.87,17.02	29	4.76	3.06,6.46	133	10.01
Education	*Elementary school or less*	423	58.75	55.14,62.35	301	49.43	45.44,53.41	724	54.48
	*Junior or senior high school*	152	21.11	18.12,24.10	145	23.81	20.42,27.20	297	22.35
	*College or above*	145	20.14	17.20,23.08	163	26.77	23.24,30.29	308	23.18
Occupation	*Officer*	111	15.42	12.77,18.07	135	22.17	18.86,25.48	246	18.51
	*Worker*	132	18.33	15.50,21.17	108	17.73	14.69,20.78	240	18.06
	*Farmer*	325	45.14	41.50,48.78	295	48.44	44.46,52.42	620	46.65
	*Others*	152	21.11	18.12,24.10	71	11.66	9.10,14.21	223	16.78
Income	*0–2000*	458	63.61	60.09,67.13	335	55.01	51.05,58.97	793	59.67
	*2001–8000*	167	23.19	20.10,26.28	176	28.90	25.29,32.51	343	25.81
	*8001 and above*	95	13.19	10.72,15.67	98	16.09	13.16,19.02	193	14.52
Hypertension	*No*	503	69.86	66.50,73.22	463	76.03	72.63,79.43	966	72.69
	*Yes*	217	30.14	26.78,33.50	146	23.97	20.57,27.37	363	27.31
CHD	*No*	601	83.47	80.75,86.19	535	87.85	85.25,90.45	1136	85.48
	*Yes*	119	16.53	13.81,19.25	74	12.15	9.55,14.75	193	14.52
Hyperlipidemia	*No*	676	93.89	92.14,95.64	573	94.09	92.21,95.97	1249	93.98
	*Yes*	44	6.11	4.36,7.86	36	5.91	4.03,7.79	80	6.02
Angina	*No*	654	90.83	88.72,92.95	577	94.75	92.97,96.52	1231	92.63
	*Yes*	66	9.17	7.05,11.28	32	5.25	3.48,7.03	98	7.37
Stoke	*No*	698	96.94	95.68,98.20	597	98.03	96.92,99.14	1295	97.44
	*Yes*	22	3.06	1.80,4.32	12	1.97	0.86,3.08	34	2.56
Diabetes	*No*	680	94.44	92.77,96.12	595	97.70	96.51,98.89	1275	95.94
	*Yes*	40	5.56	3.88,7.23	14	2.30	1.102,3.49	54	4.06
Cancers	*No*	613	85.14	82.53,87.74	562	92.28	90.16,94.41	1175	88.41
	*Yes*	107	14.86	12.26,17.46	47	7.72	5.59,9.84	154	11.59
COPD	*No*	632	87.78	85.38,90.18	567	93.10	91.08,95.12	1199	90.22
	*Yes*	88	12.22	9.82,14.62	42	6.90	4.22,8.92	130	9.78
NCDs	*No*	381	52.92	49.26,56.57	397	65.19	61.39,68.98	778	58.54
	*1*	168	23.33	20.24,26.43	108	11.73	14.69,20.78	276	20.77
	*2*	94	13.06	10.59,15.52	67	11.00	8.51,13.49	161	12.11
	*3 or more*	77	10.69	8.43,12.96	37	6.08	4.17,7.98	114	8.58

### Prevalence of NCDs

Out of 1329 participants, 363 were either previously diagnosed with hypertension or met the diagnosis criteria of hypertension, with an overall hypertension prevalence of 27.3% (30.1% in female and 24.0% in male). Fifty-four participants (4.06%) reported that they were diagnosed with diabetes before (5.56% among female and 2.30% among male), 80 participants had hyperlipidemia (6.02%). Besides these, the participants reported that 98 of them had angina (7.37%), 34 suffered from stroke (2.32%), 193 (14.52%) had CHD, 154 (11.59%) had any kind of cancers and 130 had COPD (9.78%) (Table 1).

Overall, 20.22% of the participants had one NCD (23.33% among female and 17.73% among male), 12.11% had two NCDs and 8.58% had three or more NCDs. In total, 551 participants had at least one kind of NCDs, with an overall NCD prevalence of 41.46% (47.08% for female and 34.81% for male).

**Figure 2:**
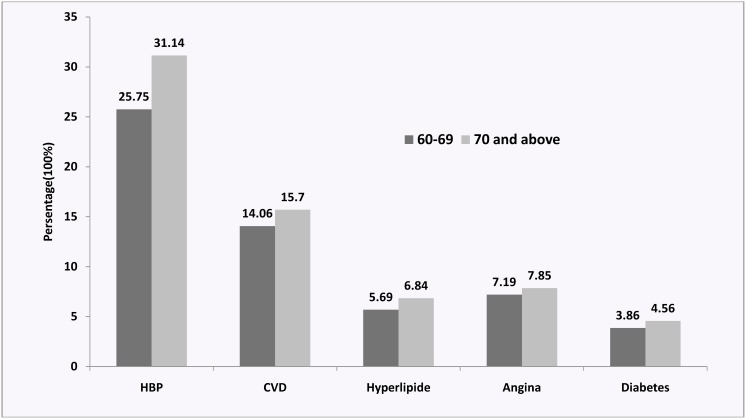
Prevalence of different kind of NCDs among different age among Uyghur elderly in Xinjiang Uyghur Autonomous Region, China (N = 1329), 2011.

Figure 2 demonstrated that the participants who were aged 70 years or more had higher prevalence of hypertension, CHD, cancer, diabetes and angina than those who were aged between 60 and 69 years.

### Behaviors and life events

The results did show that only about 18% of the participating population were not satisfied with their current life (19.0% among female and 17.1% among male), 7% were current smokers (2.5% among female and 11.5% among male), and 2–3% percent were current drinkers (0.7% among female and 4.6% among male). Majority (81.4%) had religious belief (80.0% among female and 82.3% among male). Overall, only about 18% did not have any hobbies (18.1% and 18.7% for female and male, respectively). More than 96% (95.4% for female and 97.0% for male) of the participants reported that they could take care of themselves in their daily life. About 39% of the participated elderly reported that they experienced at least one of the listed negative events in the past two years. In addition, about 13%, 16% and 40% of the participants reported that they consumed fresh vegetables, fruits and garlic two or more times per day, respectively. Only about 8% participants consumed more than 7 grams salt per day, 12% often ate chili, and 21% were still involved in farming work (Table 2).

**Table 2:** Behaviors and life events of Uyghur elderly (60 or older) in Xinjiang Uyghur Autonomous Region, China (N = 1329), 2011.

Variables		Female (n = 720)			Male (n = 609)			Total (N = 1329)	
		Frequency	Percent	95% CI	Frequency	Percent	95% CI	Frequency	Percent
Satisfaction regarding life events	*Very satisfied*	131	18.19	15.37,21.02	113	18.56	15.46,21.65	244	18.36
	*satisfied*	452	62.78	59.24,66.32	392	64.37	60.55,68.18	844	63.51
	*Not satisfied*	137	19.03	16.15,21.90	104	17.08	14.08,20.07	241	18.13
Smoking	*No*	702	97.50	96.36,98.64	539	88.51	85.96,91.05	1241	93.38
	*Yes*	18	2.50	1.36,3.64	70	11.49	8.95,14.03	88	6.62
Alcohol drinking	*No*	715	99.31	98.70,99.91	581	95.40	93.73,97.07	1296	97.52
	*Yes*	5	0.69	0.09,1.30	28	4.60	2.93,6.26	33	2.48
Religion belief	*No*	144	20.00	17.07,22.93	108	17.73	14.69,20.78	252	18.96
	*Yes*	576	80.00	77.07,82.93	501	82.27	79.22,85.31	1077	81.04
Number of hobbies	*0*	130	18.06	15.24,20.87	114	18.72	15.61,21.82	244	18.36
	*1*	126	17.50	14.72,20.28	105	17.24	14.23,20.25	231	17.38
	*2*	232	32.22	28.80,35.64	197	32.35	28.62,36.07	429	32.28
	*3 or more*	232	32.22	28.80,35.64	193	31.69	27.98,35.40	425	31.98
Have the ability to take care of themselves in daily life	*Yes*	687	95.42	93.88,96.95	591	97.04	95.70,98.39	1278	96.16
	*No*	33	4.58	3.05,6.11	18	2.96	1.61,4.30	51	3.84
Total negative events in the past two years	*No*	423	58.75	55.14,62.35	384	63.05	59.21,66.90	807	60.72
	*1*	156	21.67	18.65,24.68	114	18.72	15.61,21.82	270	20.32
	*2*	82	11.39	9.06,13.71	77	12.64	10.00,15.29	159	11.96
	*3 or more*	59	8.19	6.19,10.20	34	5.58	3.75,7.41	93	7.00
Fresh Vegetables consumption	*Two or more times per day*	108	15.00	12.38,17.61	68	11.17	8.66,13.67	176	13.24
	*One time per day*	66	9.17	7.05,11.28	41	6.73	4.74,8.73	107	8.05
	*Two or more times per week*	392	54.44	50.80,58.09	380	62.40	58.54,66.26	772	58.09
	*One time per week or less*	154	21.39	18.39,24.39	120	19.70	16.54,22.87	274	20.62
Fruits consumption	*Two or more times per day*	131	18.19	15.37,21.02	79	12.97	10.30,15.65	210	15.80
	*One time per day*	84	11.67	9.32,14.02	62	10.18	7.77,12.59	146	10.99
	*Two or more times per week*	376	52.22	48.56,55.88	362	59.44	55.53,63.35	738	55.53
	*One time per week or less*	129	17.92	15.11,20.72	106	17.41	14.38,20.42	235	17.68
Garlic Consumption	*Two or more times per day*	282	39.17	35.59,42.74	240	39.41	35.52,43.30	522	39.28
	*One time per day*	102	14.17	11.61,16.72	90	14.78	11.95,17.60	192	14.45
	*Two or more times per week*	255	35.42	31.92,38.92	220	36.12	32.30,39.95	475	35.74
	*One time per week or less*	81	11.25	8.94,13.56	59	9.69	7.33,12.04	140	10.53
Salt consumption	*3 grams or less per day*	284	39.44	35.87,43.02	216	35.47	31.66,39.28	500	37.62
	*4–6 grams per day*	378	52.50	48.84,56.16	340	55.83	51.87,59.78	718	54.03
	*7 grams or more per day*	58	8.06	6.06,10.05	53	8.70	6.458,10.95	111	8.35
Involved in farm work currently	*No*	606	84.17	81.49,86.84	442	72.58	69.02,76.13	1048	78.86
	*Yes*	114	15.83	13.16,18.51	167	27.42	23.87,30.98	281	21.14

### Correlates of NCDs

Both crude and adjusted models (adjusted for gender, education and occupation) indicated that compared to the participants who were living in urban area, the rural residents were more likely to suffer from increased no. NCDs (unadjusted Odds Ratio: OR = 1.45, 95% CI: 1.17–1.80 while adjusted Odds Ratio: AOR = 2.00, 95% CI: 1.53–2.61). In addition, both in crude and adjusted analyses, compared to those who were not satisfied with their daily life, the participants who were satisfied or very satisfied with their life had significantly lower chance of having more NCDs. Compared to those who had no religious beliefs, participants who were religious had higher odds of having more NCDs (OR = 2.75, 95% CI: 2.01–3.75; AOR in model one = 3.07, 95% CI: 2.22–4.25). Model one did also show that compared to current non-smokers, current smokers had significantly higher odds of having more NCDs (AOR = 1.53, 95% CI: 1.00–2.34) (Table 3).

**Table 3:** Factors correlated with more NCDs in ordinal logistic regression among Uyghur Elderly in Xinjiang Uyghur Autonomous Region, China (N = 1329), 2011.

Variables		Crude Model		Model 1*		Model 2#		
		OR	95% CI	OR	95% CI	OR	95% CI	
Residence	*Urban*	*Ref*		*Ref*		*Ref*		
	*Rural*	1.45	1.17,1.80	2.00	1.53,2.61	1.99	1.51,2.62	
Life satisfaction	*Not satisfy*	*Ref*		*Ref*		*Ref*		
	*Satisfy*	0.82	0.63,1.08	0.84	0.64,1.11	0.84	0.64,1.11	
	*Very satisfy*	0.59	0.41,0.84	0.61	0.43,0.88	0.62	0.43,0.89	
Alcohol Drinking	*No*	*Ref*		*Ref*		*Ref*		
	*Yes*	1.14	0.59,2.21	1.36	0.69,2.67	1.32	0.67,2.60	
Smoking	*No*	*Ref*		*Ref*		*Ref*		
	*Yes*	1.30	0.86,1.95	1.53	1.00,2.34	1.55	1.02,2.37	
Religion belief	*No*	*Ref*		*Ref*		*Ref*		
	*Yes*	2.75	2.01,3.75	3.07	2.22,4.25	3.08	2.23,4.26	
Number of hobbies	*No*	*Ref*		*Ref*		*Ref*		
	*1*	0.33	0.23,0.46	0.33	0.24,0.47	0.33	0.23,0.46	
	*2*	0.84	0.62,1.14	0.89	0.65,1.22	0.85	0.62,1.17	
	*3 or more*	0.74	0.57,0.96	0.75	0.58,0.98	0.74	0.56,0.96	
Have the ability to take careof themselves in daily life	*Yes*	*Ref*		*Ref*		*Ref*		
	*No*	4.36	2.63,7.24	4.64	2.78,7.74	4.45		2.64,7.51
Total negative events inthe past two years	*No*	*Ref*		*Ref*		*Ref*		
	*1*	1.24	0.94,1.61	1.28	0.97,1.67	1.29		0.98,1.68
	*2*	1.25	0.90,1.74	1.37	0.98,1.91	1.39		0.99,1.94
	*3 or more*	1.63	1.09,2.44	1.59	1.06,2.39	1.59		1.05,2.39
Fresh Vegetables consumption	*Two or more times per day*	*Ref*		*Ref*		*Ref*		
	*One time per day*	4.66	2.40,9.03	4.81	2.47,9.36	4.83		2.48,9.40
	*Two or more times per week*	8.75	5.091,15.04	9.45	5.48,16.32	9.45		5.47,16.34
	*One time per week or less*	12.72	7.20,22.48	12.82	7.23,22.75	12.84		7.23,22.78
Fruit consumption	*Two or more times per day*	*Ref*		*Ref*		*Ref*		
	*One time per day*	4.13	2.58,6.61	4.44	2.76,7.14	4.47		2.78,7.19
	*Two or more times per week*	3.57	2.44,5.23	3.76	2.55,5.53	3.75		2.54,5.53
	*One time per week or less*	5.43	3.54,8.32	5.40	3.50,8.34	5.42		3.51,8.37
Garlic consumption	*Two or more times per day*	*Ref*		*Ref*		*Ref*		
	*One time per day*	1.28	0.92,1.77	1.32	0.95,1.84	1.32		0.94,1.84
	*Two or more times per week*	1.52	1.19,1.94	1.55	1.19,2.01	1.56		1.20,2.02
	*One time per week or less*	1.83	1.28,2.61	1.80	1.25,2.60	1.84		1.27,2.66
Salt consumption	*Less than 4 grams per day*	1.91	1.53,2.39	1.99	1.58,2.50	2.00		1.59,2.51
	*4–7 grams per day*	*Ref*		*Ref*		*Ref*		
	*More than 7 grams per day*	1.91	1.31,2.80	1.98	1.35,2.91	1.97		1.34,2.90
Involving farm work currently	*Yes*	*Ref*		*Ref*		*Ref*		
	*No*	1.45	1.11,1.90	1.41	1.05,1.90	1.38		1.03,1.86

Note: *model 1 adjusted for gender, education and occupation;

# model 2 adjusted for gender, education, occupation, age and marital status.

The crude and adjusted model one demonstrated that not being able to take care of themselves in daily life was also positively associated with having more NCDs (OR = 4.36, 95% CI: 2.63–7.24; AOR = 4.64, 95% CI: 2.78–7.74). Not being involved in farm work currently was also found to be positively associated with suffering from more NCDs in both crude (OR = 1.45, 95% CI 1.11–1.90) and adjusted model one (AOR = 1.41, 95% CI 1.05–1.90). Both crude and adjusted model one also demonstrated that compared to those who consumed fresh vegetables more than two times per week, participants who consumed less fresh vegetables had significantly higher (for One time per day: AOR = 4.81, 95% CI 2.47–9.35; for Two or more times per week: AOR = 9.45, 95% CI 5.48–16.32 and for One time per week or less AOR = 12.82, 95% CI 7.23–22.75) chance of developing more NCDs. Similar results were observed for less fruits and garlic consumptions.

Crude and adjusted model one further pointed out that compared to those who consumed salt between 4 and 6 grams per day, participants who consumed less or more salt had higher odds of having more NCDs (AOR = 1.99 for 3 grams or less, 95% CI 1.58–2.50, and AOR = 1.98 for 7 grams or more, 95% CI 1.35–2.91).

Having one (AOR = 0.33, 95% CI 0.23–0.47), three or more (AOR = 0.75, 95% CI 0.58–0.98) hobbies were found to be negatively associated with having more NCDs, compared to these who had no hobbies (Table 3).

After further adjustment for age and marital status (model two, table 3), the results did not change much, compared to model one.

## Discussion

In this cross-sectional survey involving a comprehensive sample of Uyghur elderly residents (60 years old or more) of Xinjiang district of China, the prevalence of NCDs like hypertension (27.31%), cancers (11.59%), COPD (9.78%) and CHD (14.52%) were measured. 8.58% participants reported that they had three or more kinds of listed NCDs. Overall, the prevalence of at least one NCD among the participants was 41.46%, which was lower than the figure reported in SAGE-China Wave 1 study [9].

Corroborating with prior observations, our study also found that with increasing age, the prevalence of NCDs also increased. One potential mechanism behind this phenomenon could be that aging process might have lead to the change of hormone secretion, which in turn resulted in decline in physical and cognitive functions [18].

The participants who were aged 70 years or more had significantly higher prevalence of hypertension and this finding was similar to the result of a meta-analysis which summarized hypertension prevalence among Chinese Han population [19].

The observed overall proportion of hypertension in our study was higher than the findings of another study conducted in Xinjiang Uyghur autonomous region, which sampled participants aged between 20 and 84 in 2007 [4]. This prevalence was also higher than the age-standardized prevalence rate of hypertension among Chinese reported in 2002, which was 17.7% [20]. As hypertension was a disease associated with aging [21], the age difference may explain part of the disparity between this presented study and others. However, the reported hypertension prevalence was still very high, which could also have increased the risk of stroke, ischemic heart disease, hypertensive disease and other cardiovascular diseases [22], [23].

Several studies reported that China is experiencing an increasing epidemic of cardiovascular diseases, and CHD already became a leading cause of morbidity and mortality among Han adults in China [24], [25]. Similar to Han population, Uyghur elderly population also seemed to be under serious threat of CHD, since 14.52% participants had CHD, 7.37% experienced angina and 2.56% had stroke before. These results pointed out that targeted intervention strategies to control these diseases should be tailored for Uyghur population.

Global cancer statistics pointed out that under the effect of aging and growth of the world population, both developing and developed countries were having increasing burdens of cancers [26]. This was also true for China, since China has the world’s largest population and meets the largest challenge of aging [27]. In this presented study, 11.59% of the participating Uyghur elderly population reported that they were suffering from cancers, which further pointed out that ethnic minority populations in China are experiencing the same situation as Han population.

In addition to CVD and cancers, the participants also reported higher prevalence of diabetes and COPD. The higher prevalence of NCDs were likely to further increase the health and economic burden on the society and the risk of related complications and death [8].

Overall, 41.46% participants reported that they had at least one of the listed NCDs, 12.11% of them were suffering from two of these diseases, and 8.58% of them had three or more NCDs. Such worsening epidemic of NCDs among Uyghur elderly population seemed to be a warning signal, as NCDs already accounted for around 60% of all deaths worldwide [28]. Urgent intervention strategies need to be designed and implemented in this ethnic minority population in China.

Our results indicated that rural participants had higher chances of developing more NCDs, which corroborated with the results of another study conducted among Chinese and Indian population [29]. One study that summarized the chronic non-communicable diseases of China also reported that rural residents had higher age-standardized death rate of NCDs, particularly for CVD and COPD [4]. Lack of education, lower life standard, lack of awareness and poor access to health care could be potential reasons for this disparity. In addition, female participants had higher NCDs prevalence than male. The female partners were older could be the main reason for this phenomenon, as older age is highly related to NCDs.

Both crude and adjusted models indicated that consuming less fruits, and fresh vegetables could significantly increase the risk of developing more NCDs. Similar findings were also reported before, which revealed that low consumption of fresh fruits and vegetables and thus the lack of their nutrient biomarkers were associated with increased risk of CVD, cancer and other NCDs [30], [31]. The mechanism behind this phenomenon was probably that fresh fruits and vegetables being rich sources of dietary fiber and other human body essential elements, could lower the risk of having NCDs [31].

Alike fresh fruits and vegetables, our study also found that less garlic consumption was also highly correlated with having more NCDs. Previous studies also demonstrated that raw garlic consumption could lower the risk of cancers [9], hypertension [32] and other NCDs, while it also could bring down the cholesterol levels, in turn reducing the risk of hyperlipidemia [33].

Previous studies demonstrated that reduced salt intake could reduce the risk of hypertension, CVD and other chronic diseases [34], [35]. However, the results of our study indicated that both too much and too less salt intake could increase the risk of developing more NCDs. The detailed mechanism behind this phenomenon for low salt intake was unknown, while reverse causation could be one potential explanation for this.

Active smoking was also correlated with more NCDs acquisition, which was similar with the findings of prior studies [36]. These findings probably emphasized on the need for tobacco control programs, although Uyghur elderly population had a lower smoking rate (6.62% in our study).

Lacking the ability of taking care of themselves in daily life and not involving in farm work were also positively correlated with having more NCDs. Reverse causation could be a potential reason for this, as NCDs might have limited the mobility of the participants, which could have prevented them from farm work and taking care of themselves. Having three or more negative events in the past two years was also positively associated with development of more NCDs. One potential reason for this could be the possibility that negative events may be correlated with psychological dysfunction, and such psychological dysfunction may influence on the secretion of hormones, which may lead to or facilitate the development of NCDs [37].

Being satisfied with current life and having three or more hobbies were negatively associated with having more NCDs. These correlations may share the same reason for the negative events, but on the other side of the coin.

One study conducted in other cities in Xinjiang [38] also reported that BMI was highly correlated with blood lipids, which suggests that we should include it in our future studies.

According to our knowledge this was the first comprehensive study in Xinjiang Uyghur Autonomous Region to determine the association of NCDs with their potential predictors. By virtue of its sampling design this study was able to recruit a representative population of Uyghur elderly. The measured prevalence of NCDs as well as the observed associations of NCDs with their potential predictor can thus be extrapolated by the policy-makers for the purpose of designing appropriate targeted interventions. Large sample size, higher response rate (91.34%) and the use of ordinal logistic regression were the strengths of this presented study.

As an observational study, our study had several limitations. Because of the cross-sectional design, temporal ambiguity prevented us from drawing causal inferences based on our results and we recommend that any such interpretation should be made with caution. Vulnerability of the self-reported information, particularly for the NCDs, might lead to misclassification for both exposure and outcome. To check the problem of miss-reporting of NCDs, we used the hypertension as a surrogate, and the sensitivity and specificity for hypertension reporting were 0.84 and 0.87, respectively. Although the non-response rate was very low in our study, a small potential for selection bias still remained, which can be in either directions. In addition, the possibility of having residual confounding due to uncontrolled or unmeasured confounders was there. Also, the multiple categories of the outcome limited our ability to calculate the overall changes in absolute risk for the major risk factors for NCDs. In addition, even the social-demographic information of the participants of our study were similar to the overall Xinjiang Uygur population, we could not general our findings to other populations in China, since majority of the Uygur population in China are living in Xinjiang.

### Conclusion

Even with these limitations, we can still conclude that the prevalence of NCDs, particular for hypertension, CVD and cancers were high among Uyghur elderly population (aged 60 or more) in Xinjiang, China. More importantly, these NCDs were highly correlated with negative life events, unhealthy life styles like less fresh fruits and vegetable consumption, less garlic consumption, too less or too much salt consumption and smoking. In addition, rural area participants were more likely to develop more NCDs. To reduce the burden of NCDs, the policy makers need to implement effective intervention strategies to promote healthy life styles among the Uyghur elderly population of China.
